# Endothelin Receptor B2 (*EDNRB2*) Gene Is Associated with Spot Plumage Pattern in Domestic Ducks (*Anas platyrhynchos*)

**DOI:** 10.1371/journal.pone.0125883

**Published:** 2015-05-08

**Authors:** Ling Li, Dan Li, Li Liu, Shijun Li, Yanping Feng, Xiuli Peng, Yanzhang Gong

**Affiliations:** Key Laboratory of Agricultural Animal Genetics, Breeding and Reproduction of Ministry of Education, College of Animal Science and Technology, Huazhong Agricultural University, Wuhan, China; University of Colorado, School of Medicine, UNITED STATES

## Abstract

Endothelin receptor B subtype 2 (EDNRB2) is a seven-transmembrane G-protein coupled receptor. In this study, we investigated *EDNRB2* gene as a candidate gene for duck spot plumage pattern according to studies of chicken and Japanese quail. The entire coding region was cloned by the reverse transcription polymerase chain reaction (RT-PCR). Sequence analysis showed that duck *EDNRB2* cDNA contained a 1311bp open reading frame and encoded a putative protein of 436 amino acids residues. The transcript shared 89%-90% identity with the counterparts in other avian species. A phylogenetic tree based on amino acid sequences showed that duck EDNRB2 was evolutionary conserved in avian clade. The entire coding region of *EDNRB2* were sequenced in 20 spot and 20 non-spot ducks, and 13 SNPs were identified. Two of them (c.940G>A and c.995G>A) were non-synonymous substitutions, and were genotyped in 647 ducks representing non-spot and spot phenotypes. The c.995G>A mutation, which results in the amino acid substitution of Arg332His, was completely associated with the spot phenotype: all 152 spot ducks were carriers of the AA genotype and the other 495 individuals with non-spot phenotype were carriers of GA or GG genotype, respectively. Segregation in 17 GA×GG and 22 GA×GA testing combinations confirmed this association since the segregation ratios and genotypes of the offspring were in agreement with the hypothesis. In order to investigate the underlying mechanism of the spot phenotype, *MITF* gene was used as cell type marker of melanocyte progenitor cells while *TYR* and *TYRP1* gene were used as cell type markers of mature melanocytes. Transcripts of *MITF*, *TYR* and *TYRP1* gene with expected size were identified in all pigmented skin tissues while PCR products were not obtained from non-pigmented skin tissues. It was inferred that melanocytes are absent in non-pigmented skin tissues of spot ducks.

## Introduction

In birds, plumage color is crucial to attract opposite sex individuals for mating and to avoid predators [1,2]. Plumage coloration variants include differences in shades of basic colors (e.g. dilution), hue (e.g. black, white, yellow) and patterns (e.g. spotting, barring) [3,4]. Biosynthesis of eumelanin and pheomelanin in melanocyte is responsible for melanin-based coloration of feathers [5]. Melanocytes differentiate from undifferentiated precursors called melanoblasts, which are derived from the neural crest cells(NCC). Melanoblasts migrate from the neural crest to the epidermis and into developing feather follicles in birds [6]. To date, various genes that affect melanocyte differentiation, proliferation, migration, survival, morphology, structure and function have been shown to affect pigmentation [2,7,8].

Aberrations in development of melanocytes resulting in white spotting phenotype has been described in several species, such as dog [4], mouse [9], alpaca [10], horse [11], rabbit [12], chicken [13] and Japanese quail [14]. Up to now, endothelin 3(*EDN3*) gene and endothelin receptor B(*EDNRB*) gene have been well studied to cause pronounced white spotting phenotype [13]. Paracrine expression of *EDN3* was shown to coordinate localized coat color differences between tabby marking and wild type cats [15]. Mutations in *EDNRB* gene were found in horse with lethal white foal syndrome [8] and piebald mice [16]. Endothelin receptor B subtype 2(*EDNRB2*) gene is a paralog of the *EDNRB* gene [17]. *EDNRB2* gene encodes a seven-transmembrane domain G-protein-coupled receptor EDNRB2 and participates in melanoblast differentiation and migration [18–21]. To date, *EDNRB2* has been investigated in chicken [13,17], quail [14], fish, monotremes (platypus) [22] and frog [23], while it has been lost in therians lineages(marsupials and placentals) with the rise of the therian sex chromosomes [22]. A study by Miwa et al. [14] showed that an Arg332His amino acid change in *EDNRB2* was associated with the panda plumage color mutation in Japanese quail(*Coturnix coturnix*). Recently, Kinoshita et al. [13] reported that a Cys244Phe mutation in *EDNRB2* was associated with the *mo* ^w^ mutation, while an Arg332His mutation was associated with the *mo* mutation in chicken.

**Fig 1:**
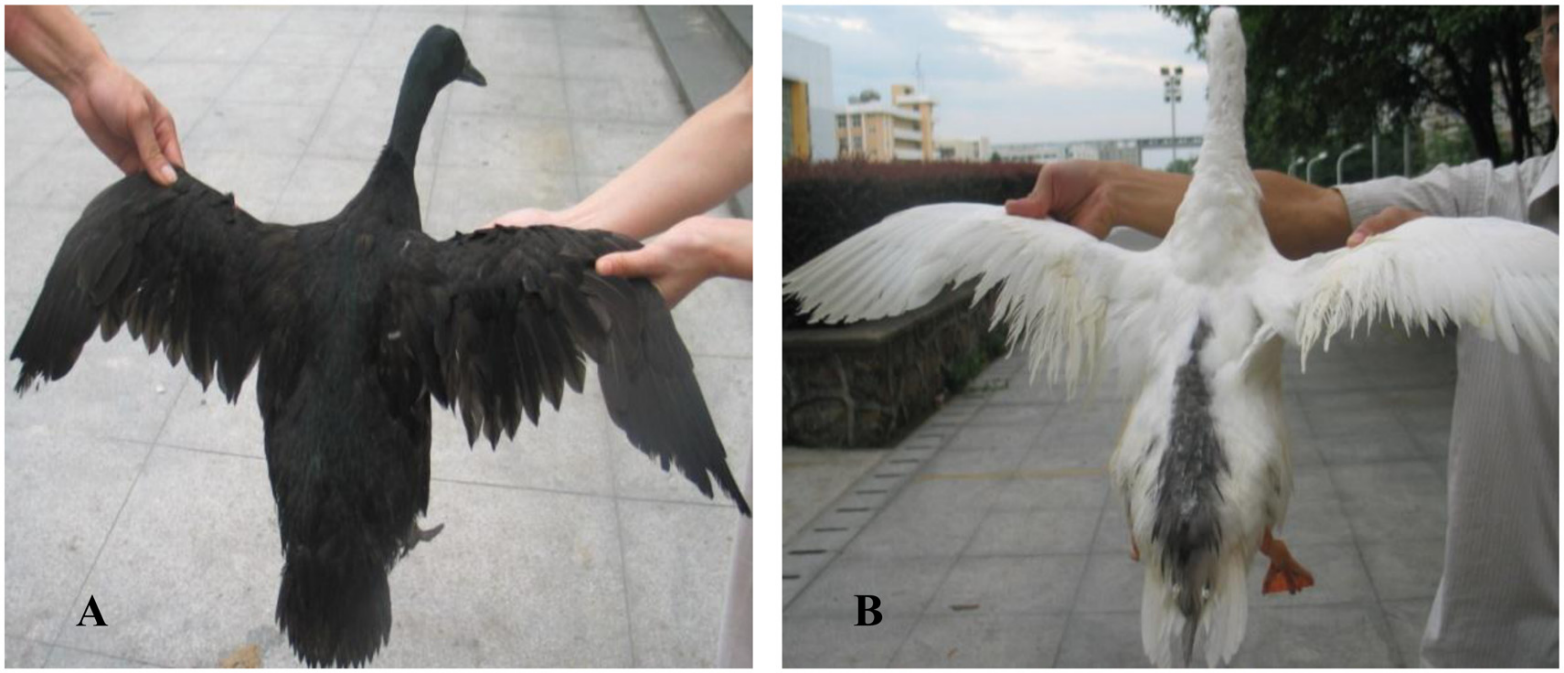
Plumage patterns of an adult non-spot(A) and spot(B) phenotype.

As described in livestock, many plumage color mutants of ducks are produced by the combined effect of controlled breeding and selection pressures from domestication [12,24]. These mutants provide opportunities for us to unravel mechanisms that controlling the inheritance of white spotting phenotype. In our previous research [24], we reported the interaction of alleles at two different loci determined the black(grey) plumage color in duck. We also found that plumage pattern of the ducks displayed great variation. Some white ducks had black patches on the head, back, tail and wing. In the current study, this kind of plumage pattern, which is similar to the panda Japanese quail described by Tsudzukis et al. [14,25] was termed as spot phenotype (Fig 1).

The aim of this study was to investigate causative mutation of the spot phenotype in duck. *EDNRB2* gene was selected as the most likely candidate gene due to its functional importance in melanocyte development and phenotypic similarity between spot duck and panda Japanese quail [14]. We characterized the transcript and genomic structure of *EDNRB2* gene in duck, performed mutation screening, investigated its association with plumage pattern, and carried out mating tests to validate the results of the association study. In addition, expression patterns of marker genes were applied to investigate the probably mechanism of the spot plumage pattern. We present strong evidence that spot plumage pattern in duck is caused by a single nucleotide substitution of the *EDNRB2* gene.

## Materials and Methods

### Ethics statement

All animal experiments were carried out according to protocols (No. 5 proclaim of the Standing Committee of Hubei People’s Congress) approved by the Standing Committee of Hubei People’s Congress, and the ethics committee of Huazhong Agricultural University, China. The approved permit number for this study is ‘‘HBAC2010113”. All efforts were made to minimize the number of animals used in this study and their suffering.

### Animals

A three-generation intercross between white *Kaiya* and white *Liancheng* has been generated in our previous study [24]. In order to verify the association between *EDNRB2* gene and spot phenotype in duck found in current research and remove the subjective bias, we deliberately designed the mating tests according to single-blind trials [26]. White ducks were used as parental ducks since their plumage pattern were not expressed due to the absence of melanin. After genotyping of the Arg332His mutation site, four mating combinations were crossed to produce 443 grey ducks: (i) two black males with genotype GA were crossed with ten white females with genotype GG, and fourteen white females with genotype GA; (ii) two white males with genotype GA were crossed with ten black females with genotype GG, and fourteen black females with genotype GA. Collection of eggs and incubation were the same as described by Gong et al. [24]. After eliminating full-sib families with less than five numbers, segregation data of plumage pattern phenotypes were analyzed with the chi-square test by the IBM SPSS Statistics Software(Version 19.0, New York, USA). All the ducks were reared in cages in a semi-open house and subjected to conventional management conditions. High quality digital pictures were taken of all one-day-old and sixty-day-old ducks (DSC-W50, Sony, Japan and ST90, Samsung, Korean) and used for phenotypic classification.

### Samples

All blood samples were collected from the F2 generation [24] comprising 647 colored ducks (non-spot and spot plumage pattern) and stored at -20°C. Tissue samples for RNA extraction were collected from skin tissues of three spot and three non-spot ducks. For each duck, six skin specimens were collected from different regions of the body (i.e., mantle, rump, breast, abdomen, the regions located at the proximal part of the wings and the distal part of the wings) after feather being plucked in each duck(S2 Fig). All skin specimens were immediately frozen in liquid nitrogen and stored at -80°C.

### DNA extraction, total RNA extraction and cDNA synthesis

DNA samples were extracted from blood samples by the phenol-chloroform method, and store at 4°C for use [27]. Total RNA was extracted from duck skin specimens using TRIzol Reagent (Invitrogen, San Diego, CA, USA) according to the manufacturer’s protocol. After RNA extraction, about 1μg DNase-treated total RNA was transcribed into cDNA using the ReverTraAce-α first strand cDNA synthesis M-MLV reverse transcriptase kit (TOYOBO, Osaka, Japan).

### Sequencing of the *EDNRB2* gene, phylogenetic analyses and identification of polymorphisms

According to the *EDNRB2* mRNA sequence of chicken and Japanese quail (GenBank accession numbers NM_204120.1, AB275309.1), we designed three pairs of primers to amplify the complete coding sequence(CDS) of duck *EDNRB2* gene by the Primer Premier 5.0 software (http://www.premierbiosoft.com). Amplification reactions were followed PCR protocols in a volume of 15 μl, using 100-200ng cDNA, 0.3μM of each primer, 10×*EasyTaq* Buffer, 2.0 mM MgSO_4_, 0.2 mM dNTPs, 0.5 unit *EasyTaq* DNA Polymerase (TransGen Biotech, Beijing, China) and ddH_2_O. Reactions were performed in a thermal cycler (Applied Biosystems, Foster City, CA, USA). PCR amplification condition was as follows: 5 min at 94°C; 35 amplification cycles of 30s at 94°C, 30s at annealing temperature, 40s at 72°C; 7 min at 72°C. PCR products were examined on 1.5% agarose gel electrophoresis and purified using the Gel Extraction Kit, then cloned into the PEASY-T1 cloning vector (TransGen Biotech, Beijing, China) and sequenced commercially (Sangon, Shanghai, China).

According to the obtained genomic sequence of duck *EDNRB2* gene, seven pairs of primers were designed to identify the polymorphisms of this gene (S1 Table). 20 spot and 20 non-spot DNA samples were applied to construct two DNA poolings, respectively. PCR products were amplified from DNA pools and sequenced commercially (Sangon, Shanghai, China). All sequences were visually edited, assembled and aligned with the SeqMan procedure of DNASTAR software (http://www.dnastar.com) and Clustal W software (http://www.ebi.ac.uk/Tools/msa/clustalw2/). Primer Premier 5.0 software was used to translate the nucleotide into protein. The protein secondary structure predictions were performed with the online website ExPASy (http://www.expasy.org/vg/index/Protein) and SMART (http://smart.embl-heidelberg.de/).

Phylogeny of endothelin receptor protein sequences was obtained with the Neighbour Joining tree option of the MEGA6.0 software [28]. All sequences used for Phylogeny and multiple sequence alignment were obtained from GenBank database. EDNRB2: Gallus gallus (NP_989451.1), Coturnix japonica (BAF42697.1), Xenopus laevis (NP_001079707.1), Myoxocephalus octodecemspinosus (ACA35037.1); EDNRB: Homo sapiens (CAM16893.1), Canis lupus familiaris (AAF81902.1), Bos taurus (DAA23820.1), Equus caballus (NP_001075306.1), Mus musculus (AAH26553.1), Coturnix japonica (CAA67681.1), Gallus gallus (AAM74023.1), Sus scrofa (NP_001033091.1), Rattus norvegicus (AEA41114.1); EDNRA: Mus musculus (AAH08277.1), Danio rerio (ABK91549.1), Xenopus laevis (NP_001080650.1), Homo sapiens (AAP32294.1), Canis lupus familiaris (BAD83849.2), Gallus gallus (AAC77793.1), Bos taurus (AAI42310.1) and EDNRC: Fundulus heteroclitus (ABY86759.1).

### Genotyping of *EDNRB2* gene and association analysis

Two pairs of primers were designed for genotyping of the 647 colored ducks of F2 generation [24] and 430 progeny of the mating tests using PCR-RFLP methods (S1 Table). PCR conditions were the same as those previously described. The restriction endonucleases NlaIII (MBI Fermentas, Hanover, MD, USA) and SfaNI (NEB, Ipswich, Massachusetts, USA) were used for the identification of the c.940G> A and c.995G> A substitution, respectively. 5 μl of PCR product was digested overnight in a standard restriction digestion protocol using 3 units of restriction enzyme at 37°C (NlaIII) and 65°C (SfaNI). Digested products were visualized on 2% (NlaIII) and 3.5% (SfaNI) agarose gel, respectively. For the c.940G>A substitution, using a PCR product amplified from genomic DNA with primer pairs NlaIII-F/R, the AA genotype is digested into two fragments of sizes 213bp and 110bp, the GA genotype is digested into three fragments of sizes 323bp, 213bp and 110bp while the GG genotype remains intact at 323bp. For the c.995G>A substitution, using a PCR product amplified from genomic DNA with primer pairs SfaNI-F/R, the GG genotype is digested into three fragments of sizes 240bp, 111bp and 23bp, the GA genotype is digested into four fragments of sizes 240bp, 134bp, 111bp and 23bp while the AA genotype is digested into two fragments of sizes 240bp and 134bp (S3 Fig). Test of significance for association between genotypes and duck plumage pattern phenotypes were conducted using Fisher′s exact test for 2×3 contigency tables by IBM SPSS Statistics Software(Version 19.0, New York, USA).

### Expression of *MITF*, *TYR* and *TYRP1* genes in skin tissues

To investigate the mechanism of the spot phenotype produced by the mutation of *EDNRB2* gene, we assessed the expression levels of three marker genes that are involved in melanocyte development. The isform M of *MITF* gene is expressed exclusively in melanocyte and required for development of NC-derived melanocyte [29–32]. Therefore, it was selected as the marker gene for melanocyte progenitor cells. The tyrosinase gene family members (e.g. *TYR*, *TYRP1*) are pigment cell-specific genes express in differentiated melanocyte [31–33]. Consequently, *TYR* and *TYRP1* gene were selected as the marker genes for melanocyte.

Four pairs of primers were designed for detecting these three marker genes and the reference gene (*β-actin*) by the Primer Premier 5.0 software. PCR reactions were carried out using primer pairs β-actin-F/R, MITF-F/R, TYR-F/R and TYRP1-F/R (S1 Table). cDNA samples of the 36 skin specimens mentioned previously were used as the template. Amplification conditions were as follows: 5 min at 94°C; 35 amplification cycles of 20s at 94°C, 20s at annealing temperature, 20s at 72°C; 5min at 72°C. All reactions contained 3 replicates. All PCR products were analyzed on a 2% agarose gel electrophoresis.

## Results

### Duck *EDNRB2* gene sequences

In our study, we amplified and sequenced 1362bp of the duck *EDNRB2* gene (GenBank: KP192480). This transcript is composed of a 1311bp open reading frame (ORF) and a 51bp 5'-untranslated region (UTR), which is 90% and 89% identical to the chicken and Japanese quail sequence (NM_204120.1, AB275309.1), respectively. The ORF of duck *EDNRB2* gene theoretically would translate into 436 amino acids (AJL35291.1), which is 95% and 95% identical to EDNRB2 of Japanese quail and chicken (BAF37676.1, NP_989451.1), respectively. The molecular weight of duck EDNRB2 is 49.02kDa and the isoelectric point(pI) is 9.01. Duck EDNRB2 is a typical membrane protein and contains seven transmembrane domains which locate at the region of 93–115, 127–149, 164–186, 206–228, 264–286, 314–333 and 353–373, respectively. Amplification products of genomic DNA were assembled and displayed a fragment of 6079bp (GenBank: KP203838) that contained the coding sequence(CDS) and intronic sequences of the *EDNRB2* gene. Exon boundaries were elucidated and numbered by comparative alignment of chicken exon sequence versus duck cDNA sequence according to the GT-AG rule.

### The phylogeny of EDNRB2

**Fig 2:**
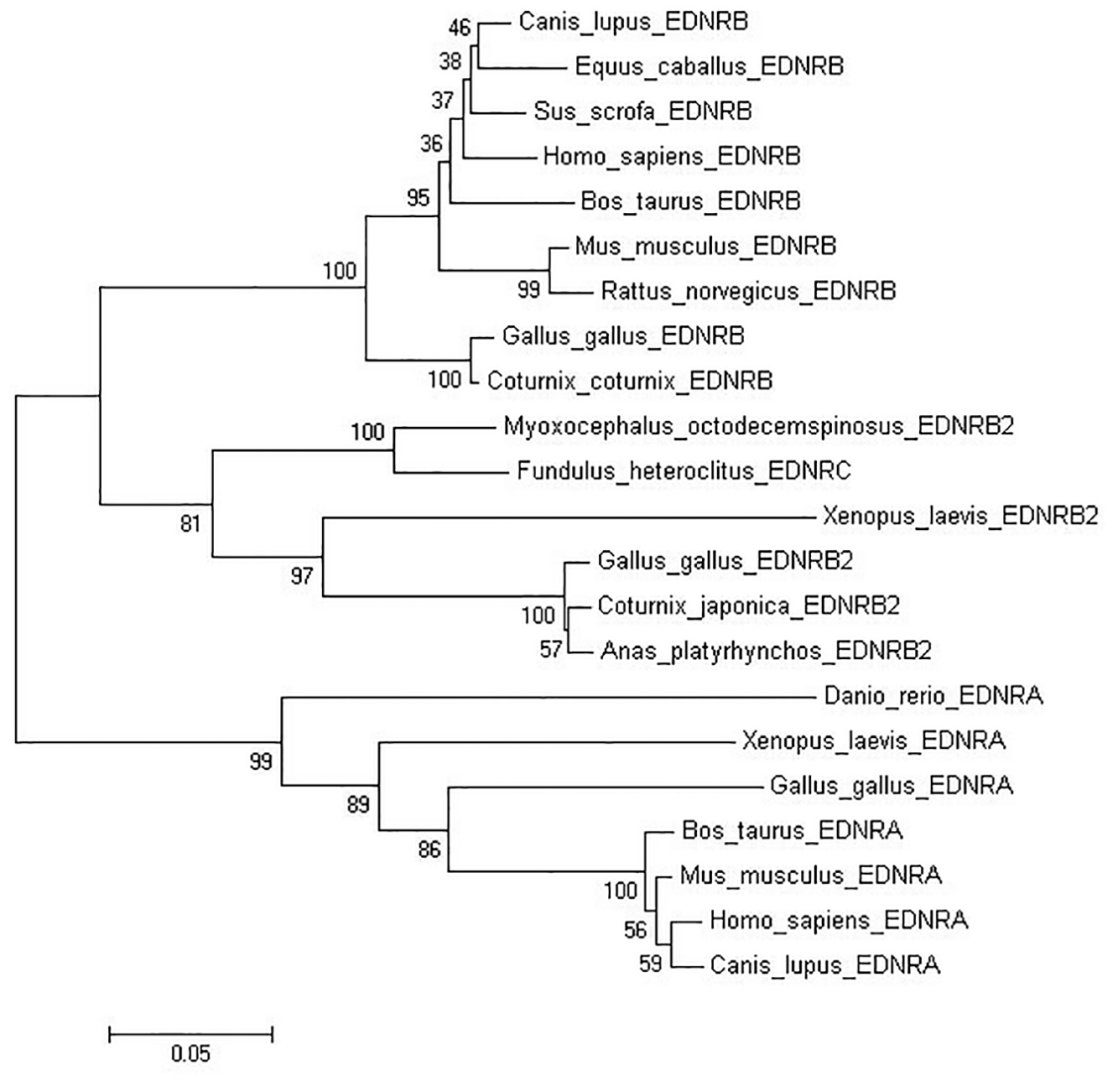
The phylogeny tree of endothelin receptors amino acid sequences. The Neighbor-Joining(NJ) method of MEGA6.0 was used to construct the phylogeny tree. The number at the branches denotes the bootstrap majority consensus values on 1000 replicates, the branch lengths represent the relative genetic distance among these species.

In order to investigate the evolutionary relationships among various species of endothelin receptors(EDNRs), a phylogeny tree was constructed based on the deduced 436aa duck EDNRB2 protein and other EDNRs. According to the results, these endothelin receptors were divided into three subgroups, EDNRB, EDNRB2(EDNRC) and EDNRA (Fig 2). This phylogeny tree indicated that the deduced duck EDNRB2 protein is closer to avian species than to the platypus, frog and fish. Duck EDNRB2 protein showed a closer phylogenic relationship with EDNRB2 than EDNRB and EDNA receptors of the mentioned species.

### Identification of polymorphisms in duck *EDNRB2* gene

**Fig 3:**
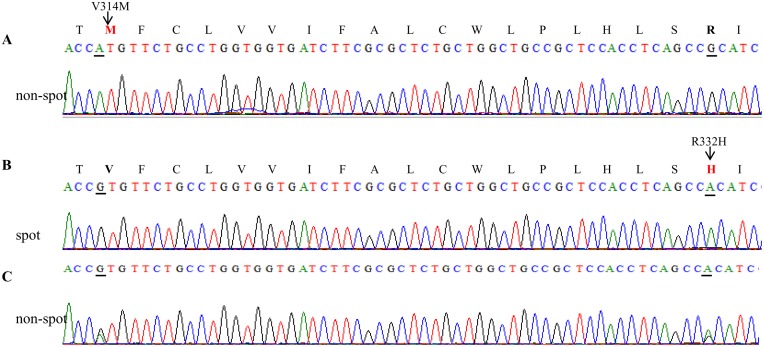
Chromatogram of *EDNRB2* DNA with different genotypes. (A) homozygous for the non-spot individuals; (B) homozygous for the spot individuals; (C) heterozygous for the non-spot individuals. The respective amino acids are shown above the second base pair for the homozygous sequences. The different amino acid are indicated in red, the amino acid sequence at the position 314 and 332 are indicated with bold fonts. The nucleotide mutations are underlined.

**Fig 4:**
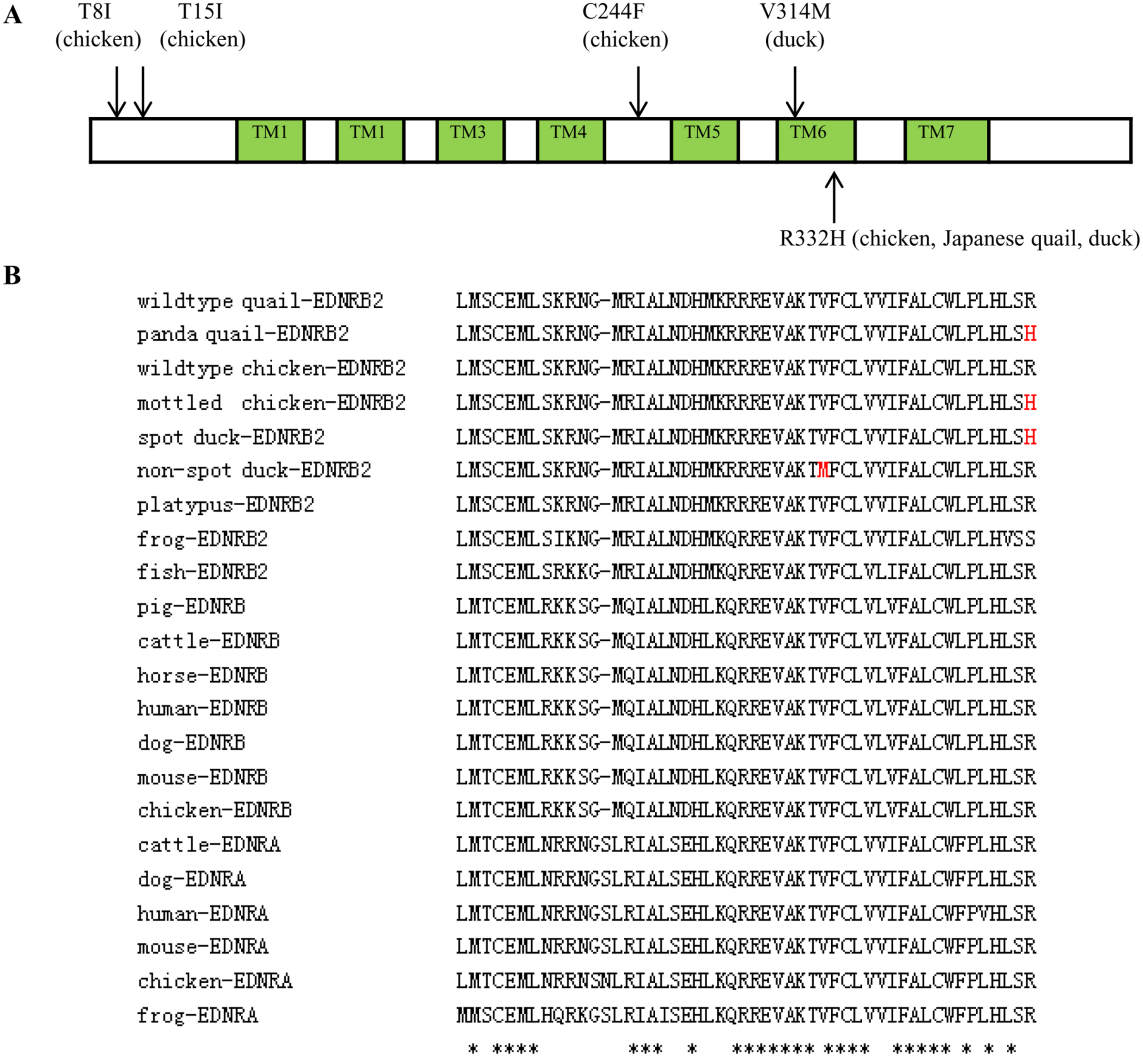
Non-synonymous substitutions in the deduced protein of duck EDNRB2. (A) Schematic diagram representing duck EDNRB2. Prediction of the secondary protein structure was obtained using online website ExPASy (http://www.expasy.org/vg/index/Protein). The extracellular and intracellular loops are shown as white boxes. The seven transmembrane domains are indicated as green boxes. Non-synonymous mutations identified in chicken [13], Japanese quail [14] and duck (in this study) are marked at the corresponding position. (B) Multiple sequence alignment of partial EDNR receptors. * Indicates amino acid identity. V314M and R332H mutations are indicated in red color.

Direct sequencing revealed 13 SNPs including 2 non-synonymous substitutions(c.940 G>A→p.Val314Met, c.995G>A→p. Arg332His) and 11 synonymous substitutions (Fig 3, S2 Table). This Arg332His mutation represents previously reported polymorphisms found in chicken [13] and Japanese quail [14]. Both non-synonymous mutations were highly conserved in endothelin receptor families and were subsequently genotyped (Fig 4).

### Association between *EDNRB2* genotypes and spot plumage pattern mutation

**Fig 5:**
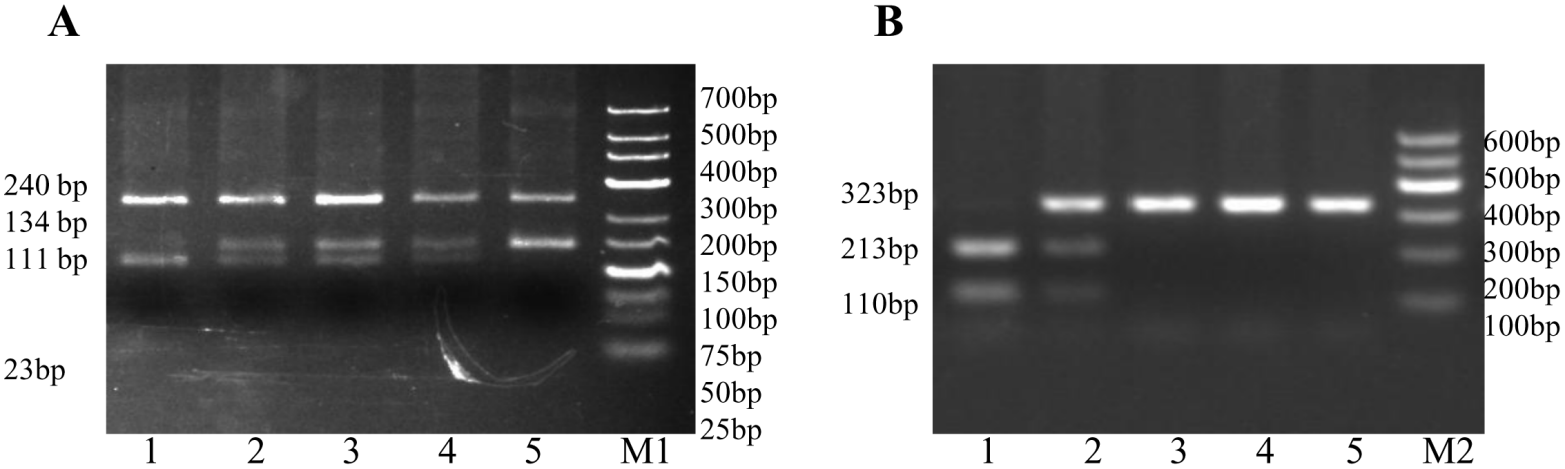
Polymorphism analyses of *EDNRB2* gene in non-spot and spot ducks. (A) SfaNI-PCR-RFLP analysis for the c.995G>A site. M1, molecular marker (low ladder); lane 1, GG genotype; lanes 2 to 4, GA genotype; lane 5, AA genotype. (B) NlaIII-PCR- RFLP analysis for the c.940 G>A site. M2, molecular marker (marker I); lane 1, AA genotype; lane 2, GA genotype; lanes 3 to 5, GG genotype.

According to the PCR-RFLP (Fig 5) and association analyses, all 152 spot individuals demonstrated a homozygous genotype AA (240/134bp) for the c.995G>A mutation, while all 495 non-spot individuals had either homozygous genotype GG (240/111/23bp) or heterozygous genotype GA (240/134/111/23bp). This mutation showed a perfect co-segregation with the spot phenotype (*P*<0.0001, Fisher′s exact test). Moreover, c.940G>A and c. 995G>A mutations were in complete linkage phase (Table 1).

**Table 1:** Association analyses between duck plumage pattern phenotypes and *EDNRB2* genotypes.

Phenotype	c. 995G>A genotype			c.940 G>A genotype		
	G/G	G/A	A/A	G/G	G/A	A/A
non-spot	134	361	-	-	361	134
spot	-	-	152	152	-	-

### Mating tests

Later on, mating tests were carried out to confirm the co-segregation of the spot locus with the c.995G>A mutation (S1 Fig). A total of 430 individuals in 39 full-sib families were analyzed (Table 2). In mating combinations GA×GG* and GA×GG^#^, it was assumed that all of the offspring would be non-spot plumage pattern. Data in Table 2 show that 191 progeny from 17 families were all non-spot plumage pattern. Thus, the segregation ratios of the non-spot and spot phenotype were in accordance with the expected ratio 1: 0. This ratio is based on the hypothesis that spot and non-spot phenotype are controlled by an autosomal allele and non-spot is dominant to spot phenotype. In mating combination GA×GA*, 80 exhibited non-spot phenotype and 25 exhibited spot phenotype. In mating combination GA×GA^#^, 98 exhibited non-spot phenotype and 36 exhibited spot phenotype. Both segregation ratios were in agreement with the expected 3:1 ration based on our hypothesis (*P*<0.80 and *P*<0.70, respectively). These results showed that the spot phenotype is recessive to the non-spot phenotype and AA genotype will show the spot phenotype.

**Table 2:** Segregation ratios of duck plumage pattern phenotypes in offspring of mating tests.

Combinations of genotypes	No. of full-sib families examined	No. of ducks examined	Phenotypes of ducks		Expected ratio of non-spot vs. spot	χ^2^ value	*P-*value
			non-spot	spot			
GA×GG*	8	88	88	-	1:0	-	-
GA×GA*	10	105	80	25	3:1	0.079	<0.80
GA×GG^#^	9	103	103	-	1:0	-	-
GA×GA^#^	12	134	98	36	3:1	0.249	<0.70

*represents white plumage drake× black plumage female duck

^#^ represents black plumage drake× white plumage female duck

All the ducks in Table 2 were genotyped to verify our hypothesis. As expected, the genotyping data of the c.995G>A and c.940G>A mutation were in accordance with our prediction. For the c.995G>A mutation, all 61 spot individuals demonstrated a homozygous genotype AA, while 171 non-spot individuals had homozygous genotype GG and 198 non-spot individuals had heterozygous genotype GA. For the c.940G>A mutation, all 61 spot individuals demonstrated a homozygous genotype GG, while 171 non-spot individuals had homozygous genotype AA and 198 non-spot individuals had heterozygous genotype GA (S3 Table).

### Expression of *MITF*, *TYR* and *TYRP1* genes in skin tissues

**Fig 6:**
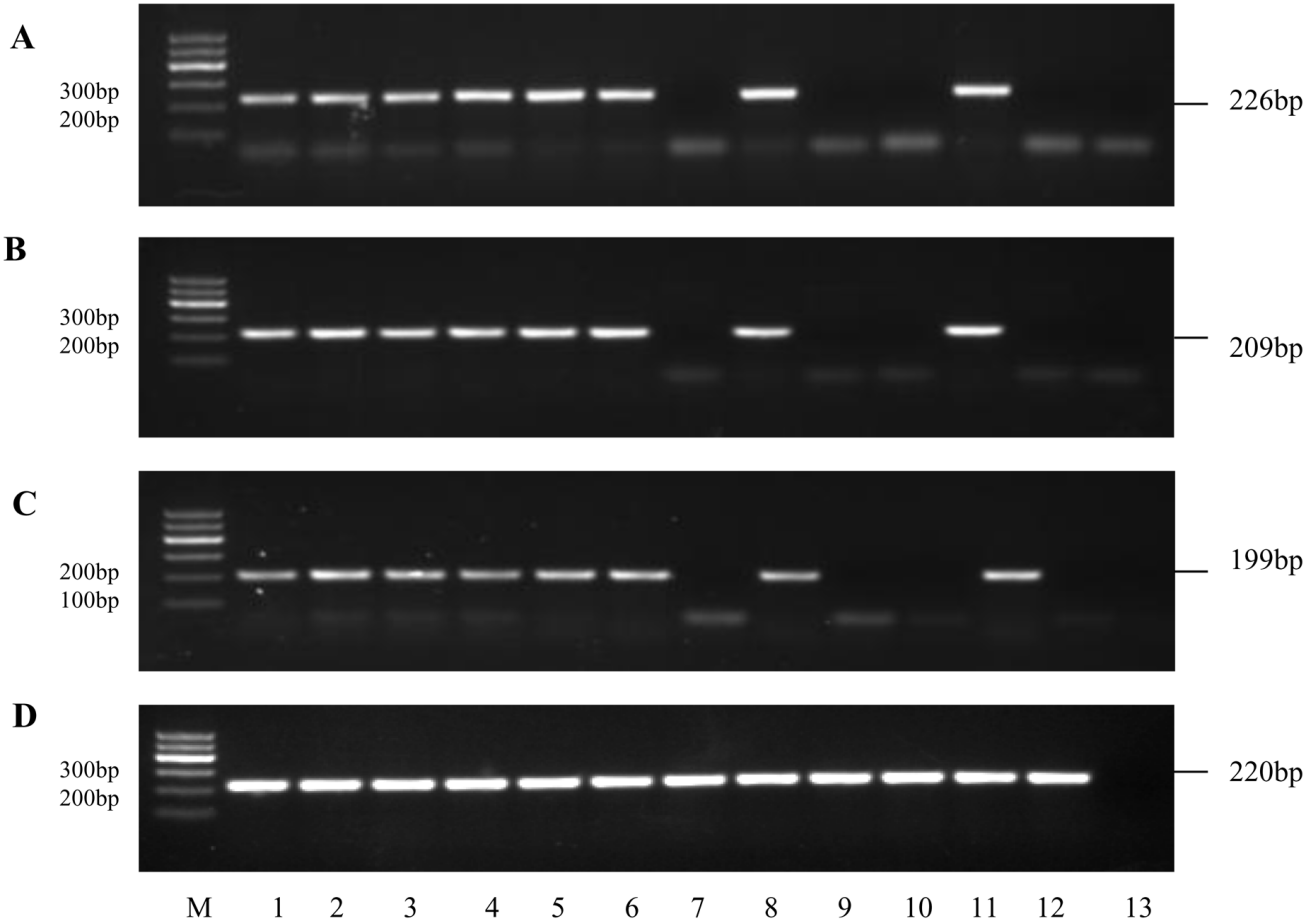
The amplified product results of duck genes. Amplification of genes from different sources, (A) *MITF* (B)*TYR* (C) *TYRP1* and (D) *β-actin* gene, Lanes 1 to 6: cDNA obtained from non-spot duck, Lanes 7 to 12: cDNA obtained from spot duck. cDNA from different area of the duck body, lanes 1 and 7: mantle; lanes 2 and 8: rump; lanes 3 and 9: breast; lanes 4 and 10: abdomen; lanes 5 and 11, the proximal part of the wings; lanes 6 and 12, the distal part of the wings; lane 13, negative control. *β-actin* gene was used as reference.

The expression patterns of the marker genes. *MITF*, *TYR* and *TYRP1* genes were found to express differentially in non-spot and spot ducks(Fig 6). Non-spot ducks (n = 3) displayed a robust *TYR*, *TYRP1* and *MITF* expression and these genes were expressed in all samples regardless of their location. These results demonstrated that both melanocyte progenitor cells and melanocytes were distributed evenly in non-spot ducks. Interestingly, *MITF*, *TYR* and *TYRP1* gene were exclusively expressed in pigmented skin tissues which located at the rump and wings in spot ducks, while they were not detected in non-pigmented skin tissues (the region located at mantle, breast, abdomen, distal part of the wings). These results indicated that both melanocyte progenitor cells and melanocytes were only found in pigmented skin tissues, while none of them were found in non-pigmented skin tissues in spot ducks. In brief, transcripts of *MITF*, *TYR* and *TYRP1* gene with expected size were identified in all pigmented skin tissues while no PCR product was obtained from non-pigmented skin tissues.

## Discussion

In this study, we reported the genomic structure of the *EDNRB2* gene, its full length transcripts in skin tissues and polymorphism of sequences between spot and non-spot ducks. The obtained nucleotide sequence shares about 89%-90% identical with *EDNRB2* gene in the chicken and Japanese quail. The phylogeny tree showed the deduced protein of duck EDNRB2 share high amino acid identities with chicken and Japanese quail. We can infer that the biological function of EDNRB2 is conservative during evolution. The closer relationship in evolution and highly conservative structure suggest that this gene may has similar functions in avian species [34,35]. The finding of *EDNRB2* gene in duck also give insight into the fact that this gene is present in aves while it was lost in the course of sex chromosome evolution in therian mammals [22].

In chicken, 4 non-synonymous mutations (Thr8Ile, Thr15Ala, Cys244Phe and Arg332His) and 5 synonymous mutations were detected [13]. In Japanese quail, 1 non-synonymous mutation (Arg332His) and 8 synonymous mutations were detected [14]. In the present study, 11 synonymous mutations and 2 non-synonymous mutations (Val314Met and Arg332His) were detected in the coding region of duck *EDNRB2*. We have not found the above synonymous mutations reported in chicken and Japanese quail, but identified 11 novel synonymous mutations [13,14]. Val314Met and Arg332His mutations were genotyped in 647 ducks representing non-spot and spot phenotypes. The valine to methionine change at the highly conserved position 314 is a novel mutation. However, this mutation is present only in non-spot ducks, so it is not responsible for the spot phenotype. We hypothesis that the Val314Met mutation has no or little influence on function of the protein. Another possible explanation is that phenotypic expression of this mutation is influenced by epistasis mutations or other related genes including *EphB2* [18,36].

Arg332His substitution of EDNRB2 has previously been reported to be associated with panda mutant in Japanese quail and mottled mutant in chicken, and it is located at the sixth transmembrane region [13,14]. In the present study, it is the most probable cause for the spot phenotype in ducks since it was only detected in all 152 spot mutant ducks of the F2 generation [24] and 61 spot ducks of the mating tests while not detected in all non-spot ducks. The results of our mating tests provided convincing evidence that this mutation was causal mutation. To date, it is the third time that the same mutation was identified in avian species and this mutational convergence suggest that evolution is highly repeatable [37]. Therefore, this mutation is highly interesting both from a genetic and a structural perspective. It is noticed that no destructive or even lethal effects of this gene were observed in duck and the identical situation is found in chicken and quail mutants [13,14]. We can speculate that the Arg332His substitution found in EDNRB2 is a neutral mutation which is not harmful for surviving or reproduction in spite of possible strong selective pressure in the wild [1,38]. Variation in *EDNRB2* sequences is able to help us to infer phylogenetic and phylogeographic relationships among organisms, assess the adaptive evolution of plumage color and understand gene evolution and evolutionary history of aves [39,40]. Although ducks diverged from the chicken approximately 90–100 million years ago[41], Arg332His substitution was found both in duck and chicken. It is postulated that this substitution of aves came from their ancestral population or introgression. This may occurred after aves’ divergence from Reptilia [37,41,42]. Further investigation of the *EDNRB2* in other 10,500 living birds and other divergent taxa (e.g. medaka, platpus) will afford opportunities to answer whether the Arg332His substitution is conserved throughout evolution [22,43].

Indeed, it seems reasonable to assume that the Arg residue at 332 position is important for protein function. The sixth transmembrane of G protein-coupled receptors (GPCRs) has been suggested to play a critical role in signaling and ligand selectivity [44,45]. We speculate that this mutation may change the 3-dimensional structure of the EDNRB2 protein(e.g. the flexibility of the third cytoplasmic loop) thus the ability of EDNRB2 to act as a EDN3 receptor is altered. One possible mechanism is that this mutation enhance the steric hindrances of the sixth transmembrane movement and reduce receptor activation [13,44,46,47]. Further genetic and pharmacological studies may help us to evaluate possible functional differences between the Arg332His change and to understand the mechanism of the convergent phenotypes caused by the Arg332His change[1,48].

The phenotypic outcomes of *EDNRB2* mutations can be partly explained based on the current understanding of its expression pattern and function. The expression of *EDNRB2* was significantly higher in Fibromelanosis skin tissue as compared to wild-type skin[49]. In panda mutant Japanese quail and mottled mutant chicken, the relative expression level of *EDNRB2* mRNA from skin was lower than in the wild type bird [13,14]. Harris et al. [18] reported that in EDNRB2-siSTRIKE-treated chicken embryos, the dorsal migration of melanoblast was reduced as a result of a severe reduction in *EDNRB2* expression. Therefore, it is postulated that normal expression level of *EDNRB2* is indispensable to normal pigmentation. The mechanism of action of the Arg332His mutation in duck remains to be established, but one possible explanation is that this mutation also down-regulates the expression of *EDNRB2* in a similar manner. Reduced expression of *EDNRB2* is supposed to cause the defective in melanocyte development in spot ducks.

In order to test our hypothesis, we used RT-PCR to investigate the expression patterns of three marker genes (*MITF*, *TYR* and *TYRP1*) in spot and non-spot ducks. Our results suggested that unpigmented areas of the spot ducks completely lack both melanocyte progenitor cells and melanocytes. Since melanocyte progenitor cells and melanocytes are derived from the melanoblasts, we hypothesize that this situation may be caused by the early elimination of melanoblasts based on other studies [32,50]. This is supported by the recent studies on the EDN3/EDNRB2 signaling which is required for melanoblast migration in *EDNRB2* gene-conserved animals. *EDNRB2* is considered to be a powerful tool for studying development of melanophore development in Xenopus and fate restrictions of premigratory avian neural crest [20,23]. In Xenopus, *EDNRB2* has an important role in melanoblast migration [23]. In chickens and quails, *EDNRB2* is upregulated in neural crest cells before the initiation of dorsolateral migration. Expression of *EDNRB2* in chick help the melanoblasts to overcome the high levels of repulsive cues in the dorsolateral environment, and it is required for melanoblasts to enter the migratory pathway and to differentiate [17–19]. Taking the evidence together, it is plausible that *EDNRB2* participates in melanocyte development in duck. In the future study, it is necessary to investigate the underlying mechanism of the lack of melanocyte in non-pigmented areas, to identify the determinants including *EDNRB2* in dorsolateral pathfinding of melanoblasts in duck and compare the results with that obtained in chicken and quail. This can be investigated by analyzing expression patterns of *EDN3*, *EDNRB2*, *MITF*, *TYR*, *TYRP1* as well as some other related genes that may be affected during the development stage of duck embryos.

Researches in the candidate genes of pigmentation are able to provide insights into human diseases, the functions and network of pigmentation related genes, and other coat color mutants with similar molecular alterations [6,13]. Given the recent advances in surgical manipulation, cell culture, cell marking techniques, transgenesis by electroporation, retrovirally mediate gene transfer and chimeras technique which have been successfully applied in study of chicken and Japanese quail embryo, rapid progress in research of duck embryo is likely [51–55]. All of the mentioned above suggest that it is indeed possible to use ducks with pigmentation mutations as a model to learn the development of melanocyte. Any mechanistic insights gained in duck could also be used to understand the role of genes involved in pigmentation.

## Conclusion

In this study, we confirmed the significant role of *EDNRB2* mutations in spot ducks and identified the causative mutation. The c.995G>A mutation results in the amino acid change Arg332His, was completely associated with the spot phenotype in duck. Our results of mating tests confirmed this association. It was inferred that melanocytes are absent in non-pigmented skin tissues of spot ducks by detected expression patterns of marker genes involved in melanocyte development. As the first study to detect polymorphism in *EDNRB2* gene of duck, the present demonstration of the spot mutation may help us to clarify the genetics mechanism of coloration in ducks. Future study may explore the functional difference between the Arg332His change and evolution of *EDNRB2* gene.

## Supporting Information

S1 Checklist Completed “The ARRIVE Guidelines Checklist” for reporting animal data in this manuscript. (DOCX)

S1 Fig Plumage patterns of spot and non-spot ducks in the mating tests. (DOC)

S2 Fig Collection of skin tissue samples used in detection of marker genes. (DOCX)

S3 Fig Schematic diagram representing SfaNI-RFLP of the PCR-amplified fragment using primer pairs SfaNI-F/R. (DOCX)

S1 Table PCR primers, PCR conditions and use of the obtained fragments. (DOCX)

S2 Table Polymorphisms identified in duck *EDNRB2* gene. (DOCX)

S3 Table Information about the full-sib families with the maximum number of offspring of the mating tests used in this study. (XLS)

## References

[1] Hubbard JK, Uy JA, Hauber ME, Hoekstra HE, Safran RJ.Vertebrate pigmentation: from underlying genes to adaptive function. Trends Genet. 2010; 26: 231–239. 10.1016/j.tig.2010.02.002 20381892

[2] Roulin A, Ducrest AL. Genetics of colouration in birds. Semin Cell Dev Biol. 2013; 24: 594–608. 10.1016/j.semcdb.2013.05.005 23665152

[3] Baxter LL, Hou L, Loftus SK, Pavan WJ. Spotlight on spotted mice: a review of white spotting mouse mutants and associated human pigmentation disorders. Pigment Cell Res. 2004; 17: 215–224. 15140066 10.1111/j.1600-0749.2004.00147.x

[4] Schmutz SM, Berryere TG. Genes affecting coat colour and pattern in domestic dogs: a review. Anim Genet. 2007; 38: 539–549. 18052939 10.1111/j.1365-2052.2007.01664.x

[5] Emaresi G, Ducrest AL, Bize P, Richter H, Simon C, Roulin A. Pleiotropy in the melanocortin system: expression levels of this system are associated with melanogenesis and pigmentation in the tawny owl (Strix aluco). Mol Ecol. 2013; 22: 4915–30. 10.1111/mec.12438 24033481

[6] Mills MG, Patterson LB. Not just black and white: pigment pattern development and evolution in vertebrates. Semin Cell Dev Biol. 2009; 20: 72–81. 10.1016/j.semcdb.2008.11.012 19073271 PMC4241852

[7] Fontanesi L, Scotti E, Colombo M, Beretti F, Forestier L, Dall'Olio S, et al A composite six bp in-frame deletion in the melanocortin 1 receptor (MC1R) gene is associated with the Japanese brindling coat colour in rabbits (Oryctolagus cuniculus). BMC Genetics. 2010; 11: 59 10.1186/1471-2156-11-59 20594318 PMC3236303

[8] Santschi EM, Purdy AK, Valberg SJ, Vrotsos PD, Kaese H, Mickelson JR. Endothelin receptor B polymorphism associated with lethal white foal syndrome in horses. Mamm Genome. 1998; 9: 306–309. 9530628 10.1007/s003359900754

[9] Bennett DC, Lamoreux ML. The color loci of mice—a genetic century. Pigment Cell Res. 2003; 16: 333–344. 12859616 10.1034/j.1600-0749.2003.00067.x

[10] Jackling FC, Johnson WE, Appleton BR.The genetic inheritance of the blue-eyed white phenotype in alpacas (Vicugna pacos). J Hered. 2014; 105: 847–857. 10.1093/jhered/ess093 23144493 PMC4201308

[11] Hauswirth R, Haase B, Blatter M, Brooks SA, Burger D, Drögemüller C, et al Mutations in MITF and PAX3 cause "splashed white" and other white spotting phenotypes in horses. PLoS Genet. 2012; 8: e1002653 10.1371/journal.pgen.1002653 22511888 PMC3325211

[12] Fontanesi L, Vargiolu M, Scotti E, Latorre R, Faussone Pellegrini MS, Mazzoni M, et al The KIT gene is associated with the english spotting coat color locus and congenital megacolon in Checkered Giant rabbits (Oryctolagus cuniculus). PLoS One. 2014; 9: e93750 10.1371/journal.pone.0093750 24736498 PMC3988019

[13] Kinoshita K, Akiyama T, Mizutani M, Shinomiya A, Ishikawa A, Younis HH, et al Endothelin receptor B2 (EDNRB2) is responsible for the tyrosinase-independent recessive white (mo(w)) and mottled (mo) plumage phenotypes in the chicken. PLOS ONE. 2014; 9: e86361 10.1371/journal.pone.0086361 24466053 PMC3900529

[14] Miwa M, Inoue-Murayama M, Aoki H, Kunisada T, Hiragaki T, Mizutani M, et al Endothelin receptor B2 (EDNRB2) is associated with the panda plumage colour mutation in Japanese quail. Anim Genet. 2007; 38: 103–108. 17313575 10.1111/j.1365-2052.2007.01568.x

[15] Kaelin CB, Xu X, Hong LZ, David VA, McGowan KA, Schmidt-Küntzel A, et al Specifying and sustaining pigmentation patterns in domestic and wild cats. Science. 2012; 337: 1536–1541. 22997338 10.1126/science.1220893 PMC3709578

[16] Yamada T, Ohtani S, Sakurai T, Tsuji T, Kunieda T, Yanagisawa M. Reduced expression of the endothelin receptor type B gene in piebald mice caused by insertion of a retroposon-like element in intron 1. J Biol Chem. 2006; 281: 10799–10807. 16500897 10.1074/jbc.M512618200

[17] Lecoin L, Sakurai T, Ngo MT, Abe Y, Yanagisawa M, Le Douarin NM. Cloning and characterization of a novel endothelin receptor subtype in the avian class. Proc Natl Acad Sci USA. 1998; 95: 3024–3029. 9501209 10.1073/pnas.95.6.3024 PMC19688

[18] Harris ML, Hall R, Erickson CA. Directing pathfinding along the dorsolateral path-the role of EDNRB2 and EphB2 in overcoming inhibition. Development. 2008; 135: 4113–4122. 10.1242/dev.023119 19004859

[19] Pla P, Alberti C, Solov'eva O, Pasdar M, Kunisada T, Larue L. Ednrb2 orients cell migration towards the dorsolateral neural crest pathway and promotes melanocyte differentiation. Pigment Cell Res. 2005; 18: 181–7. 15892714 10.1111/j.1600-0749.2005.00230.x

[20] Krispin S, Nitzan E, Kassem Y, Kalcheim C. Evidence for a dynamic spatiotemporal fate map and early fate restrictions of premigratory avian neural crest. Development. 2010;137: 585–95. 10.1242/dev.041509 20110324

[21] Nitzan E, Krispin S, Pfaltzgraff ER, Klar A, Labosky PA, Kalcheim C. A dynamic code of dorsal neural tube genes regulates the segregation between neurogenic and melanogenic neural crest cells. Development. 2013; 140: 2269–79. 10.1242/dev.093294 23615280 PMC3653553

[22] Braasch I, Volff JN, Schartl M. The endothelin system: evolution of vertebrate-specific ligand-receptor interactions by three rounds of genome duplication. Mol Bio Evol. 2009; 26: 783–99. 19174480 10.1093/molbev/msp015

[23] Kawasaki-Nishihara A, Nishihara D, Nakamura H, Yamamoto H. ET3/Ednrb2 signaling is critically involved in regulating melanophore migration in Xenopus. Dev Dyn. 2011; 240: 1454–1466. 10.1002/dvdy.22649 21538684

[24] Gong Y, Yang Q, Li S, Feng Y, Gao C, Tu G, Peng X, et al Grey plumage colouration in the duck is genetically determined by the alleles on two different, interacting loci. Anim Genet. 2010; 41: 105–108. 10.1111/j.1365-2052.2009.01967.x 19814756

[25] Tsudzuki M, Nakane Y, Wakasugi N, Mizutani M. Allelism of panda and dotted white plumage genes in Japanese quail. J Hered. 1993; 84: 225–229. 8228175 10.1093/oxfordjournals.jhered.a111325

[26] Sasaki T, Nakayama K, Yasuda H, Yoshida M, Asamura T, Ohrui T, et al A randomized, single-blind study of lansoprazole for the prevention of exacerbations of chronic obstructive pulmonary disease in older patients. J Am Geriatr Soc. 2009; 57: 1453–7. 10.1111/j.1532-5415.2009.02349.x 19515110

[27] Sambrook JF, Russell DW. Molecular cloning: a laboratory manual. 3rd ed Cold Spring Harbor Laboratory Press, 2001.

[28] Tamura K, Stecher G, Peterson D, Filipski A, Kumar S. MEGA6: Molecular Evoluti- onary Genetics Analysis version 6.0. Mol Biol Evol. 2013; 30: 2725–9. 10.1093/molbev/mst197 24132122 PMC3840312

[29] Harris ML, Erickson CA. Lineage specification in neural crest cell pathfinding. Dev Dyn. 2007; 236: 1–19. 16894594 10.1002/dvdy.20919

[30] Thomas AJ, Erickson CA. The making of a melanocyte: the specification of melanoblasts from the neural crest. Pigment Cell Melanoma Res. 2008; 21: 598–610. 10.1111/j.1755-148X.2008.00506.x 19067969

[31] Li S, Wang C, Yu W, Zhao S, Gong Y. Identification of genes related to white and black plumage formation by RNA-Seq from white and black feather bulbs in ducks. PLOS ONE. 2012; 7: e36592 doi: 10,1371//journal.pone.0036592 22615785 10.1371/journal.pone.0036592 PMC3352928

[32] Lin SJ, Foley J, Jiang TX, Yeh CY, Wu P, Foley A, et al Topology of feather melanocyte progenitor niche allows complex pigment patterns to emerge. Science. 2013; 340: 1442–1445. 10.1126/science.1230374 23618762 PMC4144997

[33] Vijayasaradhi S, Doskoch PM, Wolchok J, Houghton AN. Melanocyte differentia-tion marker gp75, the brown locus protein, can be regulated independently of tyro-sinase and pigmentation. J Invest Dermatol. 1995; 105: 113–9. 7615964 10.1111/1523-1747.ep12313414

[34] Wang C, Li SJ, Yu WH, Xin QW, Li C, Feng YP, et al Cloning and expression profiling of the VLDLR gene associated with egg performance in duck (Anas platyrhynchos). Genet Sel Evol. 2011; 43: 29 10.1186/1297-9686-43-29 21819592 PMC3162882

[35] Wang C, Li S, Li C, Feng Y, Peng XL, Gong Y. Molecular cloning, expression profile, polymorphism and the genetic effects of the dopamine D1 receptor gene on duck reproductive traits. Mol Biol Rep. 2012; 39: 9239–9246. 10.1007/s11033-012-1797-3 22740132

[36] Nadeau NJ, Minvielle F, Mundy NI. Association of a Glu92Lys substitution in MC1R with extended brown in Japanese quail (Coturnix japonica). Anim Genet. 2006; 37: 287–289. 16734695 10.1111/j.1365-2052.2006.01442.x

[37] Manceau M, Domingues VS, Linnen CR, Rosenblum EB, Hoekstra HE. Convergence in pigmentation at multiple levels: mutations, genes and function. Philos Trans R Soc Lond B Biol Sci. 2010; 365: 2439–2350. 10.1098/rstb.2010.0104 20643733 PMC2935106

[38] Gonçalves GL, Paixão-Côrtes VR, Freitas TR. Molecular evolution of the melan- ocortin 1-receptor pigmentation gene in rodents. Genet Mol Res. 2013; 12: 3230–45. 10.4238/2013.February.28.24 23479169

[39] Baião PC, Parker PG. Evolution of the melanocortin-1 receptor (MC1R) in Boob- ies and Gannets (Aves, Suliformes). J Hered. 2012; 103: 322–9. 10.1093/jhered/esr151 22351934

[40] Suzuki H. Evolutionary and phylogeographic views on Mc1r and Asip variation in mammals. Genes Genet Syst. 2013; 88: 155–164. 24025244 10.1266/ggs.88.155

[41] Huang Y, Li Y, Burt DW, Chen H, Zhang Y, Qian W, et al The duck genome and transcriptome provide insight intoan avian influenza virus reservoirspecies.Nat Genet. 2013; 45: 776–83. 10.1038/ng.2657 23749191 PMC4003391

[42] Ellegren H. Evolutionary stasis:the stable chromosomes of birds. Trends Ecol Evol. 2010; 25: 283–291. 10.1016/j.tree.2009.12.004 20363047

[43] Zhang G, Li C, Li Q, Larkin DM, Lee C, Storz JF, et al Comparative genomics reveals insights into avian genome evolution and adaptation. Science. 2014; 346: 1311–1320. 10.1126/science.1251385 25504712 PMC4390078

[44] Chen M, Cai M, McPherson D, Hruby V, Harmon CM, Yang Y. Contribution of the transmembrane domain 6 of melanocortin-4 receptor to peptide [Pro5, DNal (2')8]-gamma-MSH selectivity. Biochem Pharmacol. 2009; 77: 114–124. 10.1016/j.bcp.2008.09.023 18930713 PMC2701352

[45] Umanah GK, Huang LY, Maccarone JM, Naider F, Becker JM. Changes in conformation at the cytoplasmic ends of the fifth and sixth transmembrane helices of a yeast G protein-coupled receptor in response to ligand binding. Biochemistry. 2011; 50: 6841–54. 10.1021/bi200254h 21728340 PMC3153567

[46] Martin SS, Holleran BJ, Escher E, Guillemette G, Leduc R. Activation of the angiotensin II type 1 receptor leads to movement of the sixth transmembrane domain: analysis by the substituted cysteine accessibility method. Mol Pharmacol. 2007; 72: 182–190. 17446269 10.1124/mol.106.033670

[47] Nadeau NJ, Mundy NI, Gourichon D, Minvielle F. Association of a single-nucleotide substitution in TYRP1 with roux in Japanese quail (Coturnix japonica). Anim Genet. 2007; 38: 609–613. 18028514 10.1111/j.1365-2052.2007.01667.x

[48] Benned-Jensen T, Mokrosinski J, Rosenkilde MM.The E92K Melanocortin 1 Receptor Mutant Induces cAMP production and arrestin recruitment but not ERK activity indicating biased constitutive signaling. PLOS ONE. 2011; 6: e24644 10.1371/journal.pone.0024644 21931793 PMC3172247

[49] Dorshorst B, Molin AM, Rubin CJ, Johansson AM, Strömstedt L, Pham MH, et al A complex genomic rearrangement involving the endothelin 3 locus causes dermal hyperpigmentation in the chicken. PLoS Genet. 2011; 7: e1002412 10.1371/journal.pgen.1002412 22216010 PMC3245302

[50] Osawa, M. Melanocyte stem cells[Internet], StemBook, ed. The Stem Cell Research Community; 2009 Jun 30. 10.3824/stembook.1.46.1, Available from: http://www.stembook.org.

[51] Elena dBM, Bronner-Fraser M. Neural Crest Migration Methods in the Chicken Embryo In: Methods in Molecular Biology. vol. 294: Cell Migration: Developmental Methods and Protocols. 2005; 294: 247–267. Humana Press Inc., Totowa, N 15576917 10.1385/1-59259-860-9:247

[52] Gao J, Yuan F, Tang X, Han H, Sha J, Yuan J, et al Contribution of blastoderm cells to Japanese quail (Coturnix coturnix japonica)-Peking duck (Anas platyrhynchos) chimeras. Anim Sci J. 2011; 82: 729–734. 10.1111/j.1740-0929.2011.00905.x 22111627

[53] Griswold SL, Lwigale PY. Analysis of neural crest migration and differentiation by cross-species transplantation. J Vis Exp. 2012; 60: 3622 10.3791/3622 22349214 PMC3369633

[54] Poynter G, Huss D, Lansford R. Japanese quail: an efficient animal model for the production of transgenic avians. Cold Spring Harb Protoc; 2009: pdb.emo112 10.1101/pdb.emo112 20147007

[55] Zhang W, Rui L, Zhang J, Yu X, Yuan F, Yan L, et al Production of chimeras between the Chinese soft-shelled turtle and Peking duck through transfer of early blastoderm cells. J Exp Bio. 2013; 216: 1786–1792. 10.1242/jeb.072843 23348946

