# Supplementary material for: Endothelin Receptor B2 (EDNRB2) Gene Is Associated with Spot Plumage Pattern in Domestic Ducks (Anas platyrhynchos)
Source: PLoS One. 2015 May 8;10(5):e0125883. doi: 10.1371/journal.pone.0125883 (PMC4425580; doi:10.1371/journal.pone.0125883)

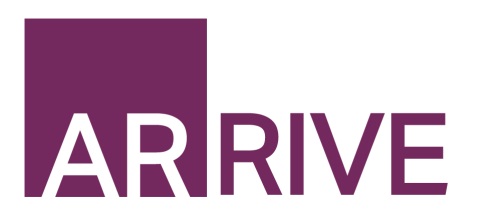


The ARRIVE Guidelines Checklist

Animal Research: Reporting In Vivo Experiments

Carol Kilkenny^1^, William J Browne^2^, Innes C Cuthill^3^, Michael Emerson^4^ and Douglas G Altman^5^

*^1^The National Centre for the Replacement, Refinement and Reduction of Animals in Research, London, UK, ^2^School of Veterinary Science, University of Bristol, Bristol, UK, ^3^School of Biological Sciences, University of Bristol, Bristol, UK, ^4^National Heart and Lung Institute, Imperial College London, UK, ^5^Centre for Statistics in Medicine, University of Oxford, Oxford, UK.*

|  | | ITEM | RECOMMENDATION | Section/ Paragraph |
| --- | --- | --- | --- | --- |
| 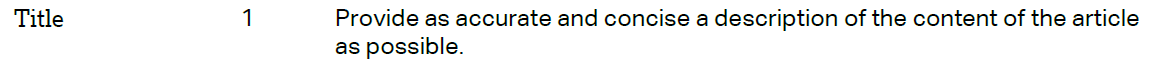 | | | Title |  |
| 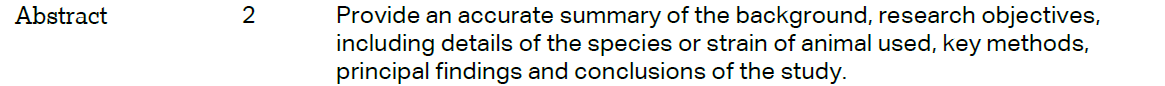 | | | Abstract |  |
| INTRODUCTION | | |  |  |
| 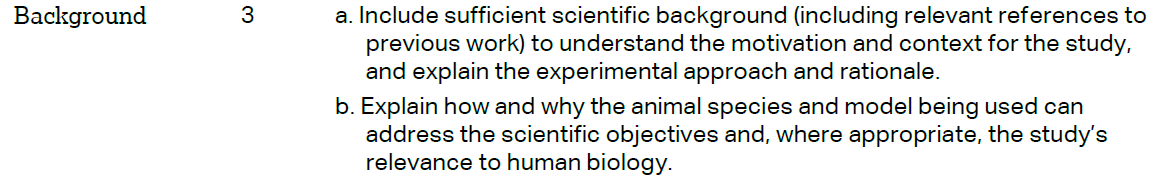 | | | Paragraphs 1-4  Paragraphs 3-4 |  |
| 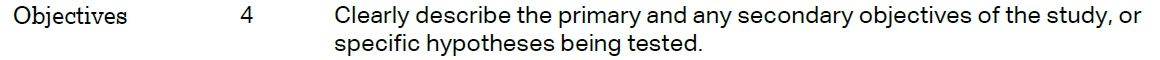 | | | Paragraph 4 |  |
| METHODS | | |  |  |
| 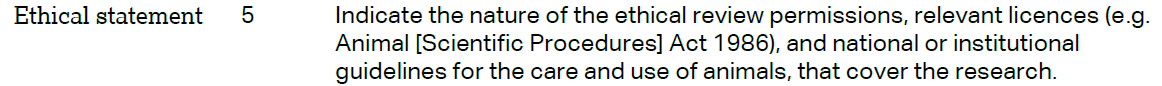 | | | Paragraph 1 |  |
| 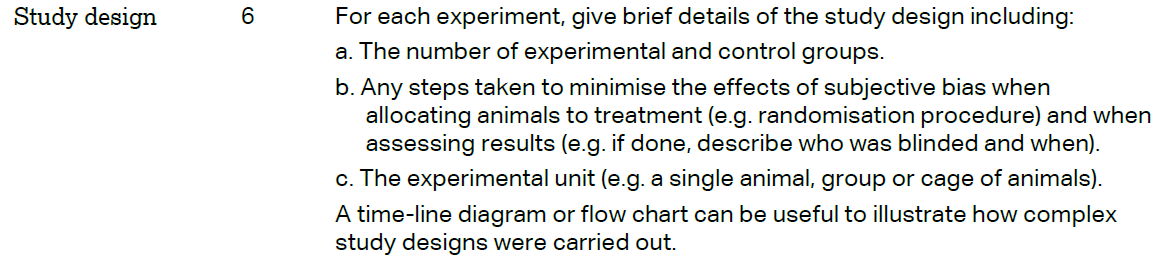 | | | Paragraphs 2,3,6-8,10  Paragraph 2  Paragraphs 2,3 |  |
|  | | | Paragraphs 2-10 |  |
| 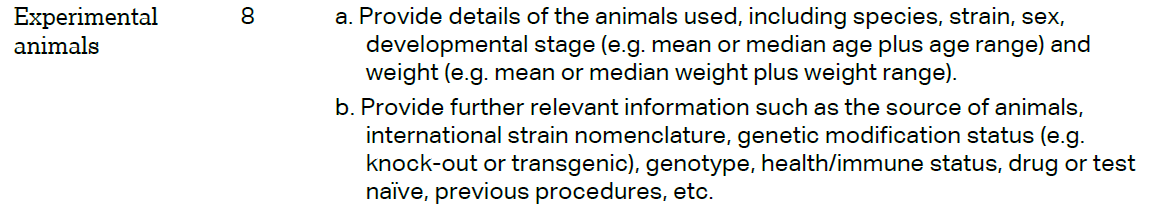 | | | Paragraphs 2,3 |  |

The ARRIVE guidelines. Originally published in *PLoS Biology*, June 2010^1^

| 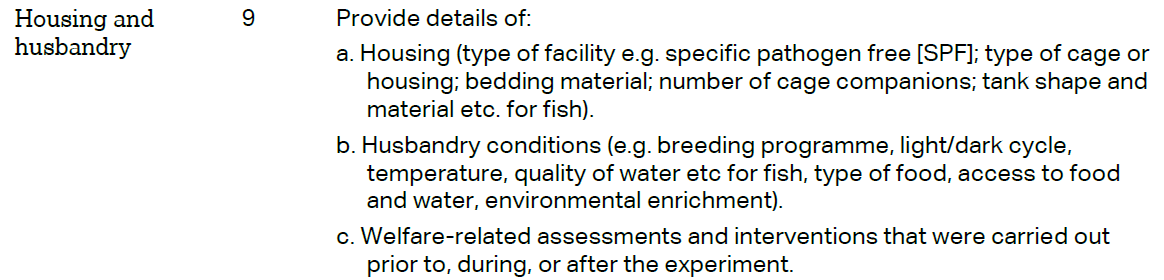 | Paragraphs1,2 |  |
| --- | --- | --- |
| 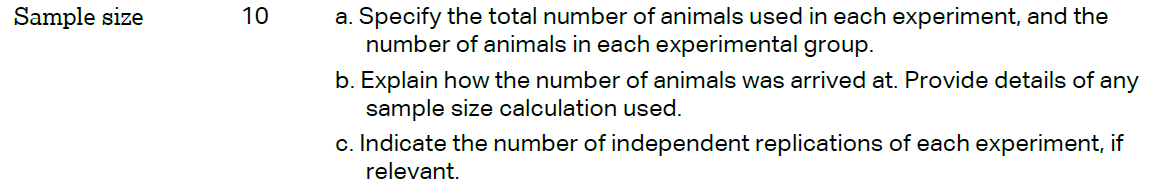 | Paragraphs 2,3,6,8,10 |  |
| 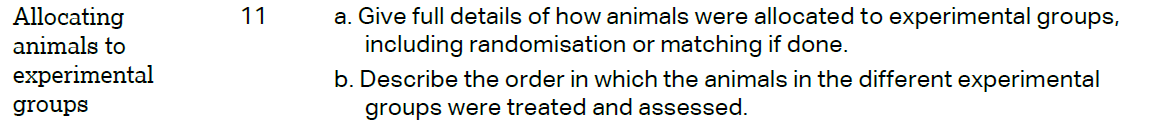 | Paragraphs 2,3 |  |
| 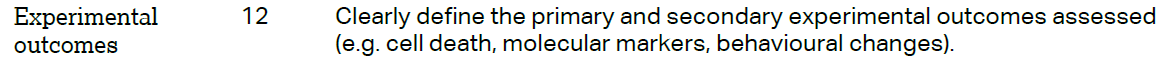 | Paragraphs 2 ,8,10 |  |
| 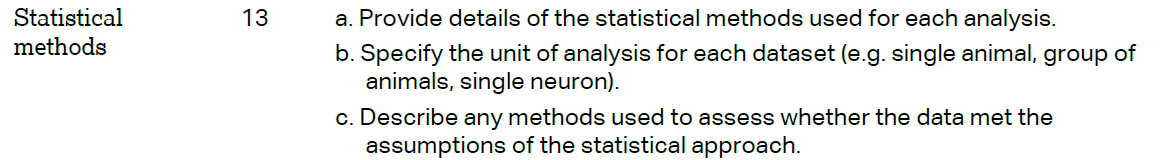 | Paragraphs 2 ,8,10 |  |
| RESULTS |  |  |
| 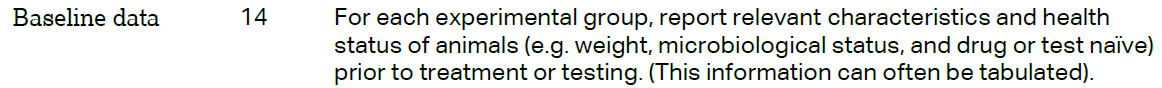 | Methods  Paragraph 2 |  |
| 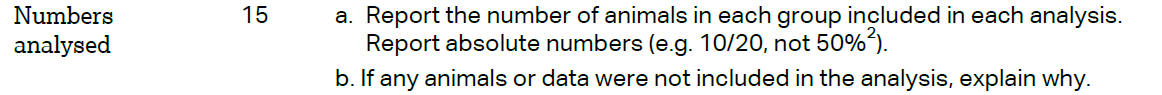 | Methods  Paragraphs  2,3,6,8,10 |  |
| 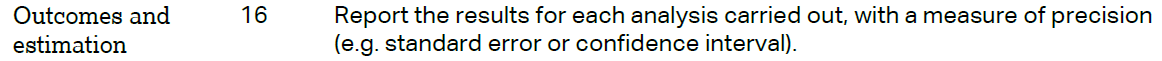 | Paragraphs 1-6 and Table 1&2 |  |
| 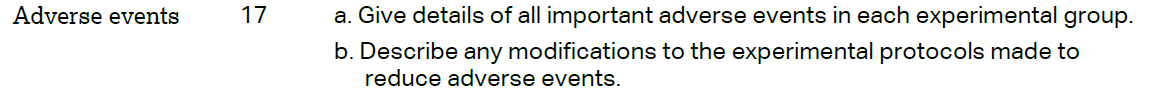 | None of adverse events existed |  |
| DISCUSSION |  |  |
| 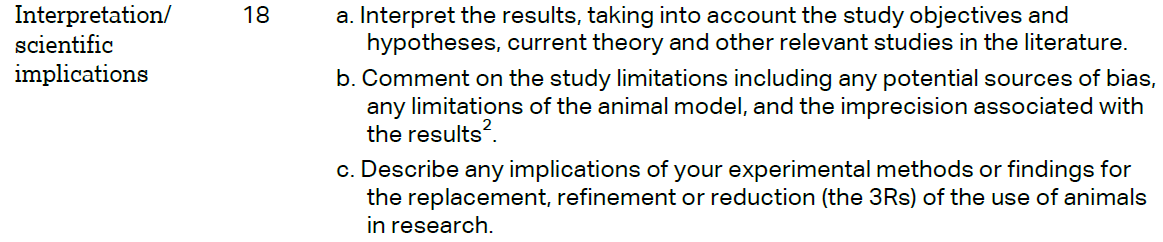 | Throughout  Paragraphs  2, 3,6  Paragraph 7 |  |
| 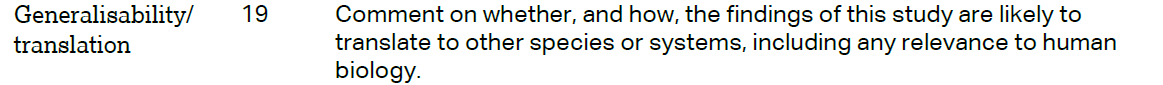 | Paragraphs 3,7 |  |
| 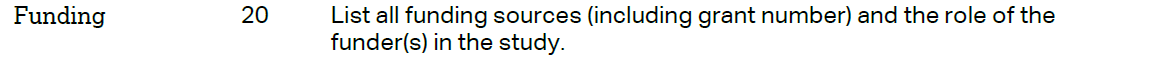 | | In financial  disclosure |


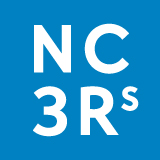

Supplement: S1 Checklist — (DOCX) [file pone.0125883.s001.docx]
