# Supplementary material for: Endothelin Receptor B2 (EDNRB2) Gene Is Associated with Spot Plumage Pattern in Domestic Ducks (Anas platyrhynchos)
Source: PLoS One. 2015 May 8;10(5):e0125883. doi: 10.1371/journal.pone.0125883 (PMC4425580; doi:10.1371/journal.pone.0125883)
Supplement: S1 Fig — (DOC) [file pone.0125883.s002.doc]

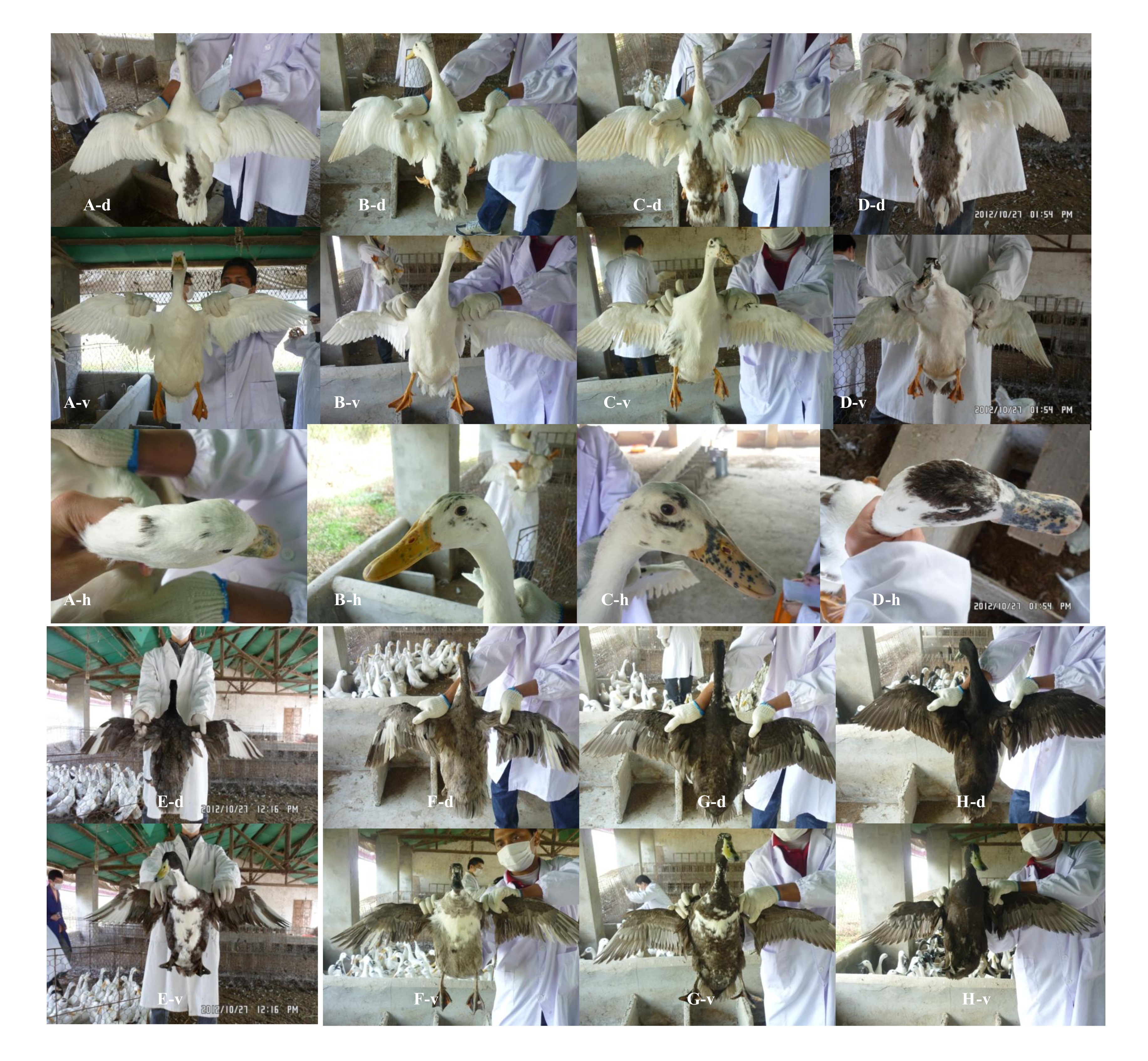


**Figure S1. Plumage patterns of spot and non-spot ducks in the mating tests.** Variation in the size of colored patch in spot and non-spot ducks. A-D: spot individuals; E-H: non-spot individuals. d:dosal; v:ventral; h:head. The proportion of colored patch area was considered as the standard of the phenotypic classification [11]. Variations of spot ducks are shown in pictures A-D. Ducks with a colored patch area on the head, back, tail and wing were considered to have a spot phenotype. The colored patch area covered about 10–35% of the body. In all 61 spot ducks, A represents the spot duck with smallest colored patch, while D represents the spot duck with largest colored patch. Variations of non-spot ducks are shown in pictures E-H. If the duck has colored patch area that exceeds colored patch area of the individual E, it was considered to have a non-spot phenotype.
