# Supplementary material for: Endothelin Receptor B2 (EDNRB2) Gene Is Associated with Spot Plumage Pattern in Domestic Ducks (Anas platyrhynchos)
Source: PLoS One. 2015 May 8;10(5):e0125883. doi: 10.1371/journal.pone.0125883 (PMC4425580; doi:10.1371/journal.pone.0125883)
Supplement: S2 Fig — (DOCX) [file pone.0125883.s003.docx]

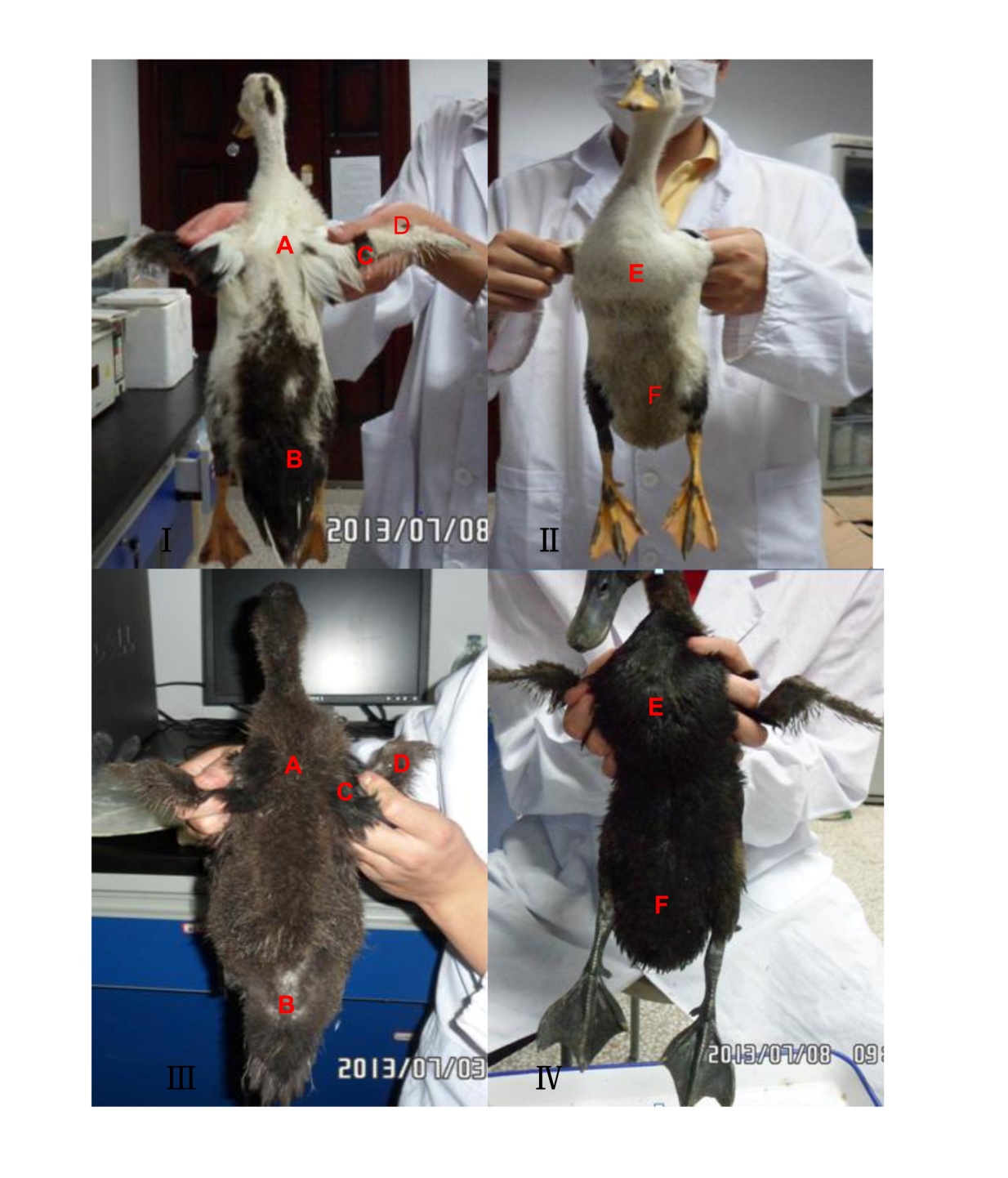


**Figure S2.** **Collection of skin tissue samples used in detection of marker genes.**

I-II: spot individual; III-IV: non-spot individual. Different area of the duck body are marked with A-F. A: mantle; B: rump; C: the proximal part of the wing(black feather); D: the distal part of the wing(white feather); E: breast; F: abdomen. In spot ducks, B and C are covered with colored feather, while A, D, E and F are covered with white feather. In non-spot ducks, A-F are all covered with colored feather.
