# Supplementary material for: Endothelin Receptor B2 (EDNRB2) Gene Is Associated with Spot Plumage Pattern in Domestic Ducks (Anas platyrhynchos)
Source: PLoS One. 2015 May 8;10(5):e0125883. doi: 10.1371/journal.pone.0125883 (PMC4425580; doi:10.1371/journal.pone.0125883)
Supplement: S3 Fig — (DOCX) [file pone.0125883.s004.docx]

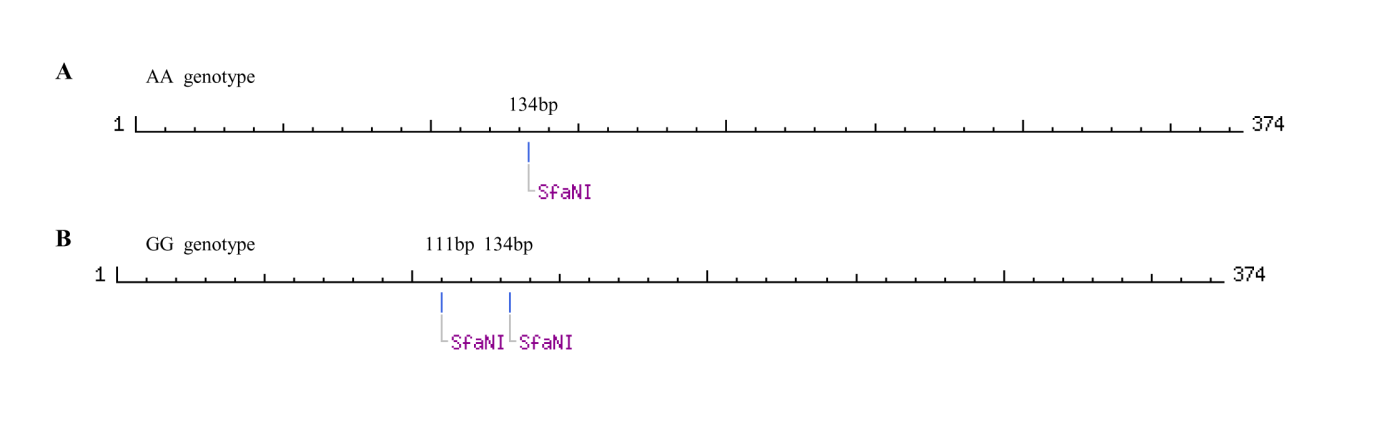


**Figure S3.** **Schematic diagram representing SfaNI-RFLP of the PCR-amplified fragment using primer pairs SfaNI-F/R.** (A) AA genotype; (B) GG genotype.
