# Supplementary material for: Endothelin Receptor B2 (EDNRB2) Gene Is Associated with Spot Plumage Pattern in Domestic Ducks (Anas platyrhynchos)
Source: PLoS One. 2015 May 8;10(5):e0125883. doi: 10.1371/journal.pone.0125883 (PMC4425580; doi:10.1371/journal.pone.0125883)
Supplement: S1 Table — (DOCX) [file pone.0125883.s005.docx]

**Table S1. PCR primers, PCR conditions and use of the obtained fragments.**

| Primer purpose | Primer name | Primer sequence(5'-3') | Product  size(bp） | Tm(°C) |
| --- | --- | --- | --- | --- |
| cDNA cloning | B2-F1 | CCCATGAGCATCCCAAGG | 596 | 62 |
|  | B2-R1 | CACAGGCTGAGTACCGTG |  |  |
|  | B2-F2 | AAGCCATAGCCTTTGACA | 721 | 56 |
|  | B2-R2 | GGATTGATGCAGGAGTTG |  |  |
|  | B2-F3 | TCAACTACTGGGACCGAGAC | 611 | 58 |
|  | B2-R3 | TTAGGAAGAGCTGTACTTG |  |  |
| Genomic DNA cloning | B2-GF1 | GAACAAGTGCATGAGGAACG | 1737 | 58 |
|  | B2-GR1 | CATAGGCTGAGCACCGTGAT |  |  |
|  | B2-GF2 | TGGTCCCCTTCATCCAGAAG | 756 | 57 |
|  | B2-GR2 | ACAGGTCTCGGTCCCAGTAG |  |  |
|  | B2-GF3 | AAGCCATAGCCTTTGACAT | 791 | 55 |
|  | B2-GR3 | AGCCACCAGTCCTTCACG |  |  |
|  | B2-GF4 | GGCATCTTCTACACCCTC | 1549 | 55 |
|  | B2-GR4 | TGGAAGCAGTTCTTGAAT |  |  |
|  | B2-GF5 | TGAATGTGGGAGCATCAAAG | 1270 | 55 |
|  | B2-GR5 | TTAGGAAGAGCTGTACTTG |  |  |
| Polymorphisms | B2-SF1 | TTTGCCGCTCAGTCCTCCAT | 482 | 58 |
|  | B2-SR1 | CGCACGAAACCCACCTTG |  |  |
|  | B2-SF2 | TGGGCTTCTTGCACCTTG | 309 | 57 |
|  | B2-SR2 | ACAGAGGGGGTTATGCTGTG |  |  |
|  | B2-SF3 | TGTTGGTGGCTCTTCTCACT | 406 | 60 |
|  | B2-SR3 | CTTTGCCTAATGCTGCTCTC |  |  |
|  | B2-SF4 | GGCATCTTCTACACCCTC | 325 | 55 |
|  | B2-SR4 | ATCTGTCCGCTCTTCACGCT |  |  |
|  | B2-SF5 | TGAGTCGTCCTCCTGAAGC | 272 | 59 |
|  | B2-SR5 | TACCTGAGCAGTTCGCATCT |  |  |
|  | B2-SF6 | TGAATGTGGGAGCATCAAAG | 291 | 62 |
|  | B2-SR6 | TGGAAGCAGTTCTTGAAT |  |  |
|  | B2-SF7 | CATTTCCAAGGCTCACCA | 344 | 54 |
|  | B2-SR7 | TTAGGAAGAGCTGTACTTG |  |  |
| Marker gene detection | β-actin-F | AACCCCAAAGCCAACAGAGAG | 220 | 58 |
|  | β-actin-R | CACGGCCAGCCAGATCCAGAC |  |  |
|  | MITF-F | TGCAGTCACTTCTCTCACAACC | 226 | 62 |
|  | MITF-R | CCAGGCGGCATGACATGATCAC |  |  |
|  | TYR-F | AATGACAAATCAAGGACTCC | 209 | 54 |
|  | TYR-R | GAGCCATTCATGTAGATGTG |  |  |
|  | TYRP1-F | TGAGATGTTTGTTACTGCACC | 199 | 58 |
|  | TYRP1-R | AGTGAGAAGAGGCTGATGCAA |  |  |
| PCR-NlaIII–RFLP | NlaIII-F | TGAGTCGTCCTCCTGAAGC | 323 | 56 |
|  | NlaIII-R | TACCTGAGCAGTTCGCATCT |  |  |
| PCR-SfaNI–RFLP | SfaNI-F | TCCCCTGCCCACCTTCTC | 374 | 60 |
|  | SfaNI-R | TCCTTTTGGCCCTGCTGA |  |  |
