# Supplementary material for: Endothelin Receptor B2 (EDNRB2) Gene Is Associated with Spot Plumage Pattern in Domestic Ducks (Anas platyrhynchos)
Source: PLoS One. 2015 May 8;10(5):e0125883. doi: 10.1371/journal.pone.0125883 (PMC4425580; doi:10.1371/journal.pone.0125883)
Supplement: S2 Table — (DOCX) [file pone.0125883.s006.docx]

**Table S2.** **Polymorphisms identiﬁed in duck *EDNRB2* gene.**

| cDNA position﹠ | Nucleotide polymorphism | Amino acid polymorphism﹡ | Restriction enzyme site﹟ |
| --- | --- | --- | --- |
| 57 | C>T | - | -- |
| 348 | C>T | - | FaiI/PsiI/SgeI |
| 354 | C>T | - | -- |
| 454 | C>T | - | EcoRII/MvaI/PstNI |
| 465 | C>T | - | ArsI/ Bse1I/LpnPI |
| 477 | C>T | - | BstC8I |
| 516 | A>G | - | HgaI/Hin1I/Hpy99I |
| 543 | G>T | - | -- |
| 861 | C>T | - | -- |
| 868 | C>T | - | BseMII/BspCNI/DdeI |
| 940 | G>A | V>M | NlaIII/Bst4CI/CviAII |
| 995 | G>A | R>H | SfaNI/BspACI/TauI |
| 1062 | C>T | - | BseRI/Hpy188III |

﹠+1 corresponds to the first nucleotide of the *EDNRB2* gene coding sequence

﹡A dash (-) indicates that no amino acid change occurred

﹟ Two dashes(--) indicate that no restriction enzyme site was found
